# Automatic skin disease diagnosis using deep learning from clinical image and patient information

**DOI:** 10.1002/ski2.81

**Published:** 2021-11-25

**Authors:** K. A. Muhaba, K. Dese, T. M. Aga, F. T. Zewdu, G. L. Simegn

**Affiliations:** ^1^ Biomedical Imaging Unit School of Biomedical Engineering Jimma Institute of Technology Jimma University Jimma Ethiopia; ^2^ Department of Biomedical Engineering Kombolcha Institute of Technology Wollo University Dessie Ethiopia; ^3^ Department of Dermatology and Venereology Jimma Institute of Health Sciences Jimma University Jimma Ethiopia; ^4^ Department of Dermatovenereology Boru‐meda Hospital Dessie Ethiopia

## Abstract

**Background:**

Skin diseases are the fourth most common cause of human illness which results in enormous non‐fatal burden in daily life activities. They are caused by chemical, physical and biological factors. Visual assessment in combination with clinical information is the common diagnostic procedure for diseases. However, these procedures are manual, time‐consuming, and require experience and excellent visual perception.

**Objectives:**

In this study, an automated system is proposed for the diagnosis of five common skin diseases by using data from clinical images and patient information using deep learning pre‐trained mobilenet‐v2 model.

**Methods:**

Clinical images were acquired using different smartphone cameras and patient's information were collected during patient registration. Different data preprocessing and augmentation techniques were applied to boost the performance of the model prior to training.

**Results:**

A multiclass classification accuracy of 97.5%, sensitivity of 97.7% and precision of 97.7% has been achieved using the proposed technique for the common five skin disease. The results demonstrate that, the developed system provides excellent diagnosis performance for the five skin diseases.

**Conclusion:**

The system has been designed as a smartphone application and it has the potential to be used as a decision support system in low resource settings, where both the expert dermatologist and the means are limited.

## Graphical abstract

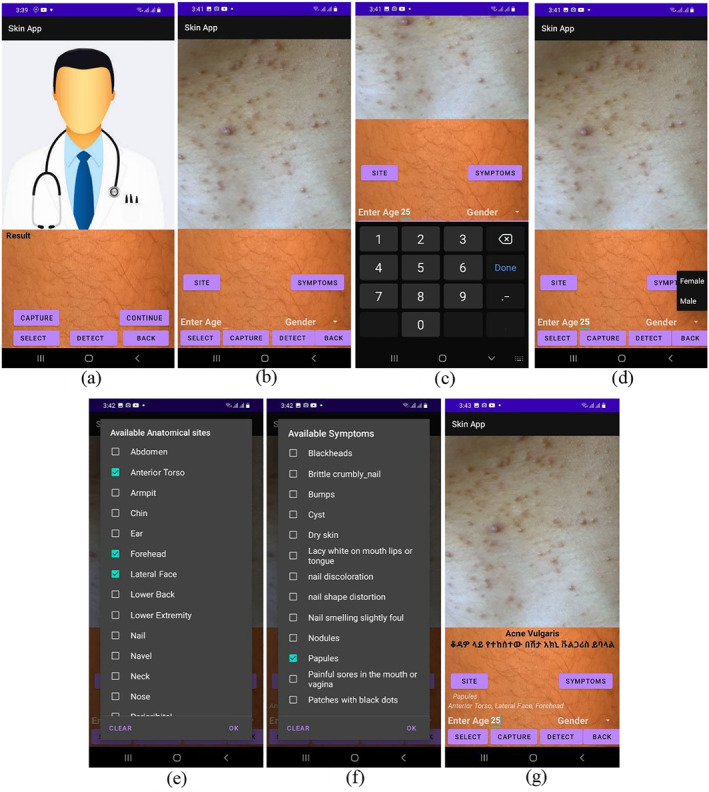

1 **What is already known about this topic?**

- The manual skin disease diagnostic procedures are time consuming, require experience and excellent visual perception, and are prone to error.

2 **What does this study add?**

- In this study, deep learning‐based automatic system is proposed for diagnosis of five common skin diseases by using data from clinical images and patient information.

## 1 INTRODUCTION

Skin is the largest organ of the body which provides protection, regulates the body fluids and temperature, and enables sense of the external environment.^1^ Skin diseases are the most common cause of all human illnesses which affects almost 900 million people in the world at any time.^2^ According to the global burden of disease project, skin disease is the fourth leading cause of non‐fatal disease burden throughout the world.^3^ An estimated 21%–87% of children in Africa are affected by skin diseases.^4^ Skin disease can cause financial, socio‐economic, and psychological burden to the community and place a strain on health professionals.^5^, ^6^, ^7^, ^8^, ^9^, ^10^, ^11^, ^12^ Moreover, skin diseases may cause a sense of depression, frustration, isolation, and even suicidal ideation.^13^

The pattern of skin diseases varies due to environmental factors, hygienic standards, social customs, and genetics. In developing countries, infection and infestation are more common.^4^ There are more than 3000 known skin diseases worldwide.^14^ According to a preliminary study conducted for this research, acne vulgaris, atopic dermatitis, lichen planus, onychomycosis and tinea capitis are among the common skin diseases in Ethiopia. A statistics show that, atopic dermatitis affects 20% of children below the age of two.^5^ Acne scarring is a long‐term complication that can affect 95% of patients with acne vulgaris.^8^ The global prevalence of onychomycosis is 5.5% and contributes 50% of all nail diseases.^15^ In Ethiopia, 32.3% of school‐aged children suffer from tinea capitis.^16^

The common procedures for diagnosing skin diseases are patient history and symptoms analysis, skin scraping, visual inspection, dermoscopic examination and skin biopsy. However, these diagnosis methods are tedious, time‐consuming, and prone to subjective diagnosis. Most of them require experience and excellent visual perception of dermatologist. Sophisticated and robust medical imaging modalities can also be used for skin disease diagnosis.^17^ However, these techniques are complex, expensive and limited to centralized healthcare facilities that leave low resource setting populations without access to dermatological service.

Recently, smartphone‐based imaging and sensing platforms have become an alternative means of disease diagnosis in the healthcare industry. The latest generation of a smartphone with a high‐definition camera, large storage capacity and high‐performance processor enables to capture of digital images and record videos with better resolution.^18^ Portability, cost‐effectiveness and connectivity make a smartphone to be applicable in many areas.^19^, ^20^ The availability of smartphones equipped with digital cameras enables the acquisition of clinical images for investigation using computer‐aided diagnosis (CAD).

CAD can reduce the burden of health care professionals with the help of artificial intelligence.^21^ Different literatures have proposed a means of diagnosing skin diseases using clinical images.^22^, ^23^, ^24^, ^25^, ^26^, ^27^, ^28^, ^29^ A support vector machine (SVM) with quadratic kernel has been proposed by Hameed et al.^24^ for classification of acne, eczema, psoriasis, benign and malignant melanoma with an accuracy of 83%. Similar accuracy (about 81%) has been claimed by Nasr‐Esfahani et al.^22^ for classification of melanoma and benign lesions using convolutional neural network. Additionally, a multiclass classification system were proposed using ResNet152 for 12 skin diseases.^25^, ^26^ Fujisawa et al.^23^ applied a pre‐trained GoogLeNet to classify 14 categories of skin tumours and an overall accuracy of 76.5% was claimed. Recently, Wu et al.^29^ compared five pre‐trained deep learning frameworks for the diagnosis of six facial skin conditions from a clinical image and using an InceptionResNet_V2 a precision of 77% was claimed. Velasco et al. proposed MobileNet CNN to classify seven skin diseases and claimed an overall accuracy of 94.4%.

The proposed works showed promising results for the diagnosis of different skin diseases from clinical images. However, most of the works were dependent on the availability of an online public dataset, focused on cancer and tumours and are designed to diagnose specific parts of a body. Moreover, the datasets collected and used mainly consists of white skin. Moreover, the diagnostic performance including the accuracy reported are not satisfactory.

In this study an automatic diagnosis system has been developed based on deep learning model for five most common skin diseases including acne vulgaris, atopic dermatitis, lichen planus, onychomycosis and tinea capitis by combining clinical images acquired using a smartphone camera and patient information.

## 2 MATERIALS AND METHOD

**FIGURE 1:**
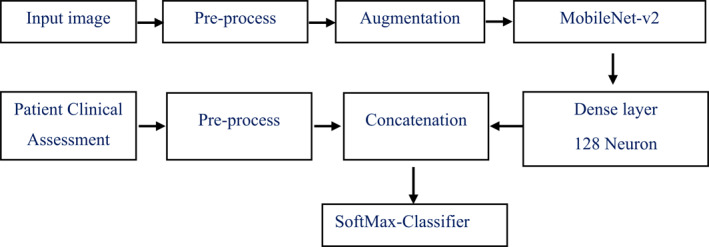
Block diagram of the skin disease multi‐class classification system

This automated diagnosis system was developed by using a pre‐trained mobilenet‐v2 model. Both skin images and patient clinical information were pre‐processed and concatenated for classification of skin diseases. Figure 1 demonstrates the general block diagram of the proposed system.

### 2.1 Data collection

**FIGURE 2:**
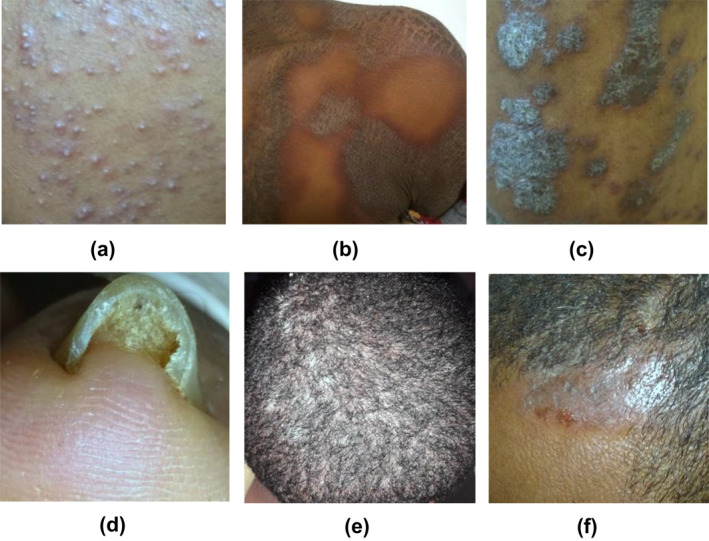
Sample image from five skin diseases (a) Acne vulgaris, (b) atopic dermatitis, (c) lichen planus, (d) onychomycosis, (e) tinea capitis and (f) seborrhoeic dermatitis (labelled as unknown class)

The dataset used for this research were collected from Dr. Gerbi medium clinic of Jimma and Boru‐meda General Hospital of Dessie from 286 patients (149 females and 119 males, age range 0–85 years). A total of 1137 images along with patient information was collected from Dr. Gerbi medium clinic and 239 images from Boru‐Meda General hospital using a smartphone camera (Nokia window phone, Techno Spark4, SamsungA20, and SamsungJ6). About 300 of the images were collected from healthy skin and 1376 from abnormal skin affected by acne vulgaris, atopic dermatitis, lichen planus, onychomycosis and tinea capitis. The images were captured after the diagnosis was confirmed by expert dermato‐venerologist and a tropical dermatologist. Moreover, images from other less common skin diseases were also included, labelled as an unknown class to reduce the false‐positive result of the model. The unknown class includes 204 images of lichen simplex chronicus, cow pox, monkey pox, leishmania, tinea corporis, rosacea, seborrhoeic dermatitis, foot ulcer, papular urticaria, discoid lupus erythematosus, onchocerciasis, real world object images and others. Table 1 shows the number of images collected for each skin diseases. Patient information including age, gender, anatomical sites and symptoms of the diseases were also collected during. The anatomical sites include; abdomen, anterior torso, armpit, chin, ear, forehead, lateral face, lower back, lower extremity, nail, neck, periorbital region, posterior torso, scalp and upper extremity. The medical sign and symptoms of the five skin diseases were also included. A total of 41 features from patient information were extracted and used to develop the model. Figure 2 demonstrates sample of collected abnormal skin.

**TABLE 1:** Data collected from Jimma and Dessie

Diseases	Number of images
Healthy	300
Acne vulgaris	307
Atopic dermatitis	300
Lichen planus	289
Onychomycosis	211
Tinea capitis	269
Unknown	204
Total	1880

**FIGURE 3:**
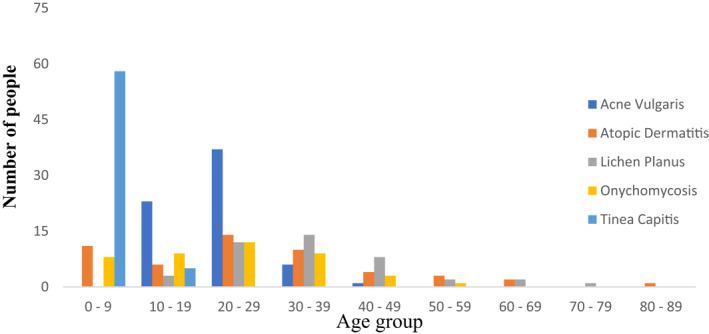
Age‐wise distribution of five skin diseases

**FIGURE 4:**
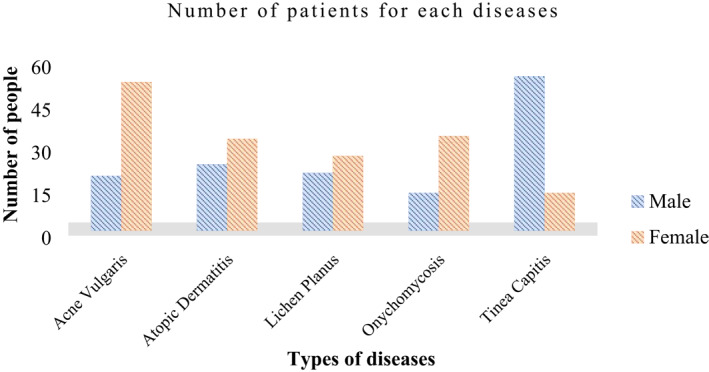
Gender‐wise distribution of the five skin diseases

Figures 3 and 4 demonstrate age wise and gender wise distribution of collected data for the selected five skin conditions. Table 2 shows the common symptom lists and anatomical sites of the five skin diseases. Table 3 demonstrates the number of clinical images collected form each anatomical site for the five skin disease.

**TABLE 2:** Common symptoms and site invaded by the five skin diseases^30^, ^31^

Diseases	Acne vulgaris	Atopic dermatitis	Lichen planus	Onychomycosis	Tinea capitis
Symptoms	Blackheads	Dry skin	Thickened nail	Nail discolouration	Scaly grey or reddened areas
	Whiteheads	Scale	Purplish flat bumps	Nail shape distortion	Patches with black dots
	Papules	Bumps	Lacy white on mouth lips or tongue	Nail smell foul	
	Pimples	Thickened cracked skin	Painful sores in the mouth or vagina		
	Nodule				
	Cysts				
Anatomical sites	Lateral face	Neck	Lower extremity	Nail	Scalp
	Forehead	Lower extremity	Upper extremity		
	Posterior torso	Upper extremity	Lower back		
	Anterior torso	Anterior torso	Neck		
	Chin	Posterior torso	Abdomen		
	Neck	Lower back	Anterior torso		
		Periorbital	Lateral face		
		Armpit	Nose		
		Lateral face	Ear		
		Forehead	Scalp		
			Armpit		
			Forehead		

**TABLE 3:** Number of clinical images collected from each anatomical site for the five skin disease

Anatomical sites	Number of clinical images collected on each site				
	Acne vulgaris	Atopic dermatitis	Lichen planus	Onychomycosis	Tinea capitis
Upper extremity	‐	107	77	‐	‐
Lower extremity	‐	63	99	‐	‐
Periorbital	‐	6	2	‐	‐
Armpit	‐	11	7	‐	‐
Navel	‐	2	‐	‐	‐
Lower back	‐	4	9	‐	‐
Scalp	‐	‐	1	‐	269
Nail	‐	‐	‐	211	‐
Abdomen	‐	‐	4	‐	‐
Nose	‐	‐	3	‐	‐
Ear	‐	‐	1	‐	‐
Lateral face	183	45	26	‐	‐
Forehead	61	4	13	‐	‐
Anterior torso	23	23	26	‐	‐
Posterior torso	31	11	4	‐	‐
Chin	5	‐	‐	‐	‐
Neck	4	24	17	‐	‐
Total	307	300	289	211	269

### 2.2 Pre‐processing

Image resizing, colour constancy, and data augmentation were performed before feeding the image to the deep learning network. All the images were resized to 224 × 224 pixels to match the input size of the pre‐trained mobilnet‐v2 model. The shades of grey colour constancy algorithm were used in the pre‐processing step to remove the colour bias of the clinical images. This was found to improve the classification accuracy of multisource images in literatures.^32^, ^33^ The dataset was split into training (80%), validation (10%) and testing (10%) prior to model training. Then data augmentation was applied to the training dataset by 90° rotation, horizontal and vertical image flipping to increase the number of datasets. The patient information was converted to a feature vector using one‐hot encoding method.

### 2.3 Repurposing pre‐trained mobilenet‐v2 model

MobileNet‐v2 was introduced by Sandler et al. in 2019,^34^ as performance improvement of mobile models. It is based on an inverted residual structure where the input and output of the residual block are thin bottle neck layers opposite to traditional residual models. The architecture of mobilenet‐v2 contains fully convolutional layer with 32 filters followed by 19 residual bottlenecks. The fully convolution operation is replaced by depth‐wise separable convolution that splits into two separable layers. First, depth‐wise convolution performs light‐weight filtering using 3 × 3 kernel per input channel. Following the depth‐wise convolution, the point‐wise convolution builds feature by computing linear combination of the input images. The feature extractor outputs 1280 image feature maps to the classifier. The model is suitable for resource limited environments including smartphones.

In this study, we have applied transfer learning approach using the pre‐trained mobilenet‐v2 model for skin disease classification. For both binary and multi‐class classification of skin diseases, using image data alone the output of the pre‐trained model was flattened and fed to the classifier. The classifier then uses the concatenation of both the image data and patient information to classify the skin disease. Since, the image data from pre‐trained mobilenet‐v2 model is larger than the patient information feature, a dense layer with 128 neurons was added at the top of the pre‐trained model. This reduces the output image features of the model to 128 and balances the two inputs types of the classifier (a one‐hot encoded patient information features and the image features). Moreover, weighted loss function based in labels frequency, which assigns more weight to less represented class, was applied to mitigate class imbalance in the dataset. Best result was found by using Adam optimized, cross entropy loss function and a learning rate of 0.0001 for binary and multiclass classification.

The performance of the model was using accuracy, precision, recall, F1‐score, and kappa score Moreover, receiver operator characteristic (ROC) curve, a graph which gives information about how the model correctly classifies positive and negative samples, and the kappa value, a metric used to compare an observed accuracy with an expected accuracy or random chance, were used as model evaluation metrics.

## 3 RESULTS

### 3.1 Pre‐processing

**FIGURE 5:**
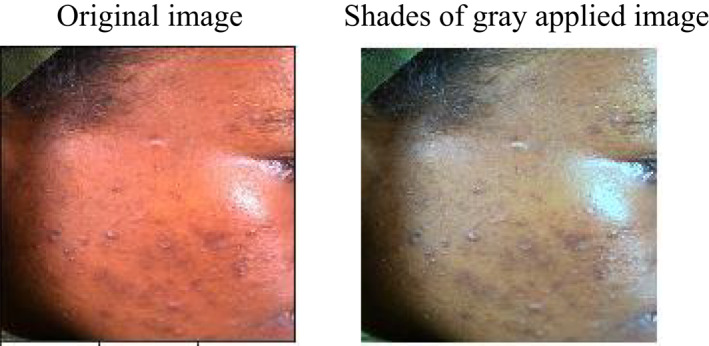
Sample result of applying shades of grey colour constancy algorithm

The clinical images were acquired using different smartphone camera under different illumination sources. The colour variation resulting from different illumination sources were estimated and corrected by applying the shades of grey algorithm. Figure 5 depicts the effect of applying shades of grey algorithm on the clinical images.

### 3.2 Result of binary classification task

**FIGURE 6:**
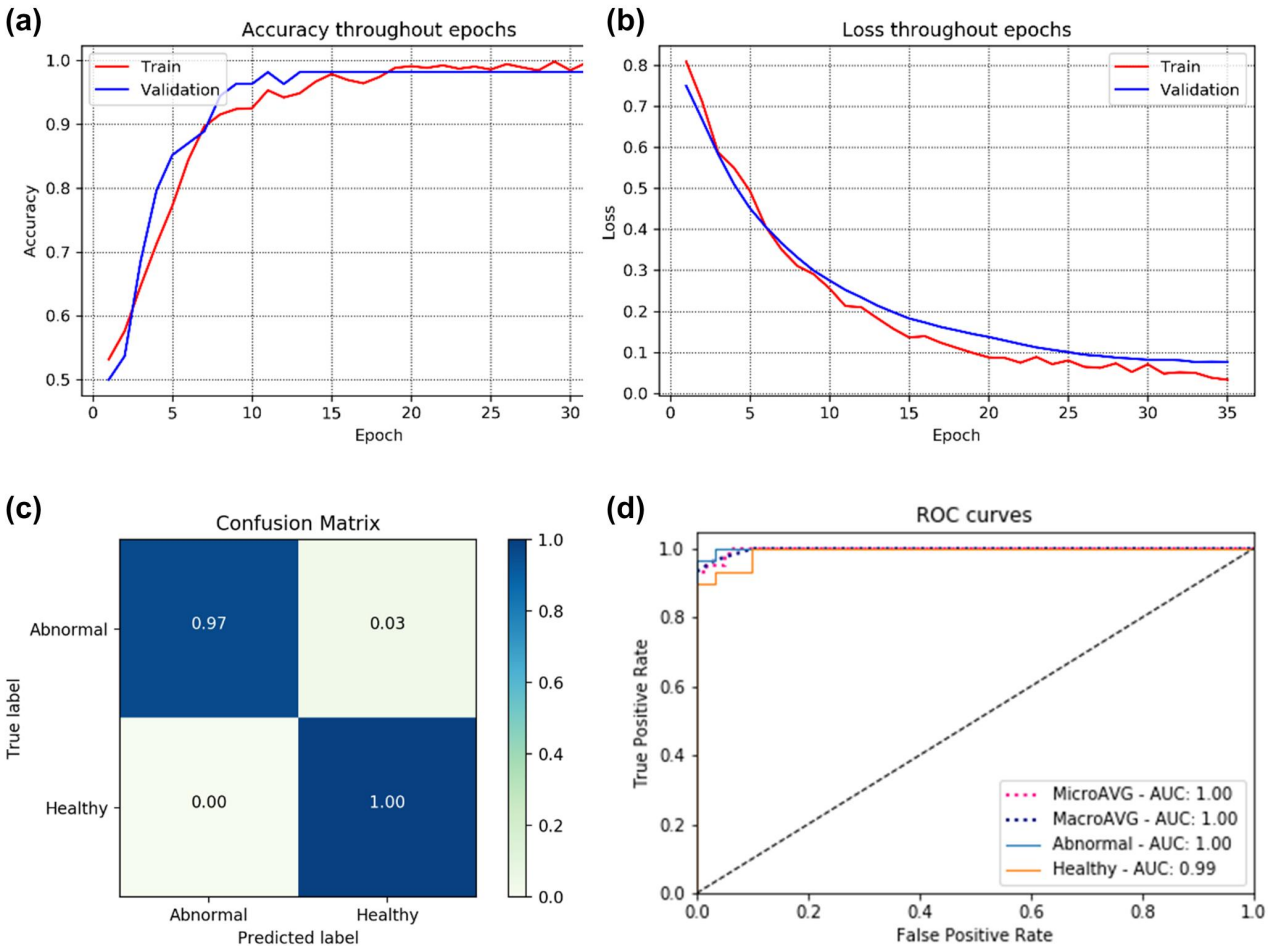
Detail of binary classifier (a) training and validation accuracy, (b) training and validation loss, (c) confusion matrix and (d) receiver operator characteristic (ROC) curve

For the binary classification task (normal and abnormal), the model correctly predicts 59 of the 60 unseen test images. The accuracy, precision, recall, F1 score and kappa values of 98.3%, 98.5%, 98.5%, 98.0% and 0.97, respectively, were achieved for binary classification. Figure 6 shows the training and validation accuracy curve, training and validation loss curve, confusion matrix and ROC curve of the binary classifier.

### 3.3 Multi‐class classification

**FIGURE 7:**
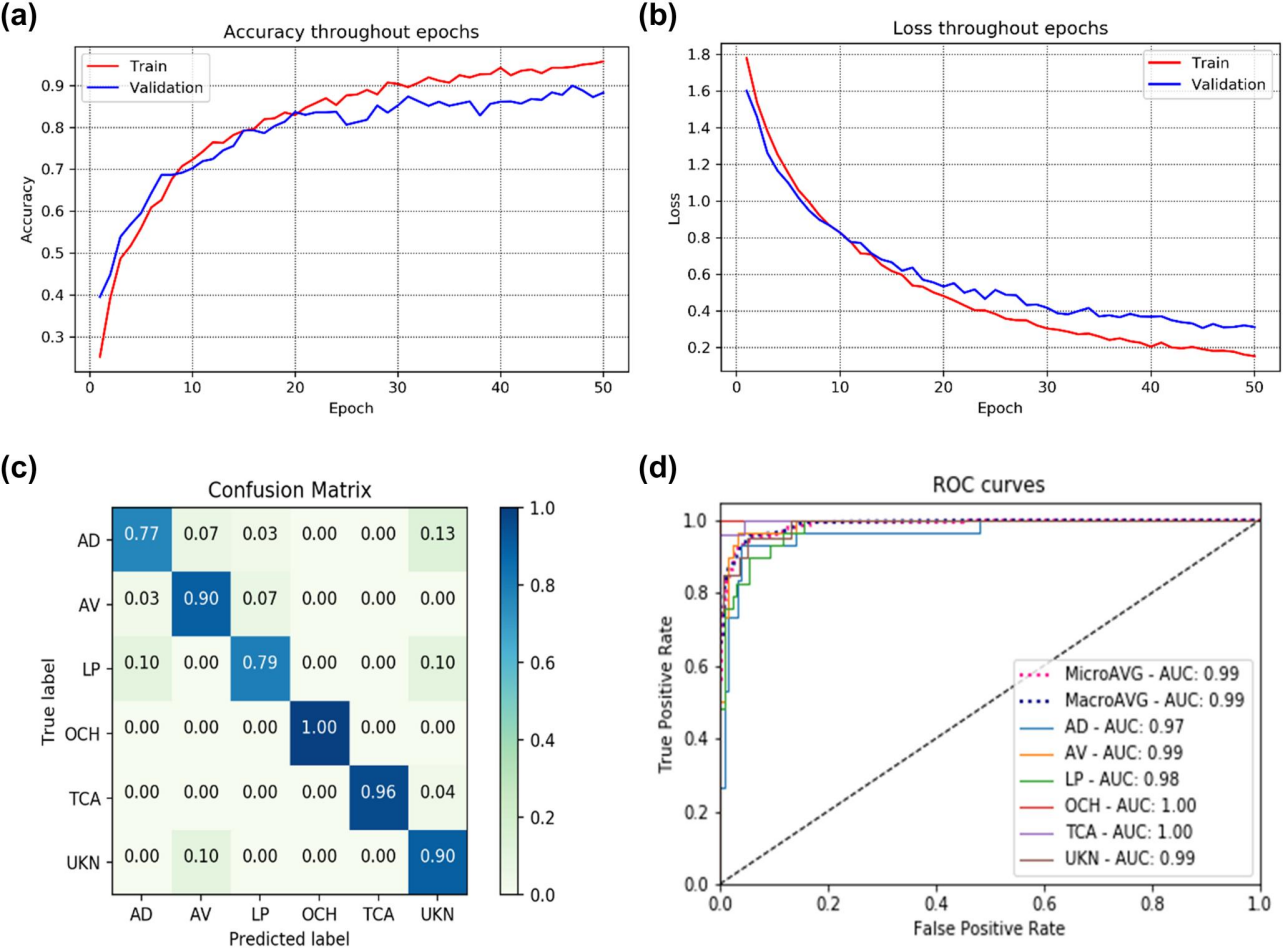
Summary of a multi‐class classifier using image only (a) training and validation accuracy, (b) training and validation loss, (c) confusion matrix and (d) receiver operator characteristic (ROC) curve

**FIGURE 8:**
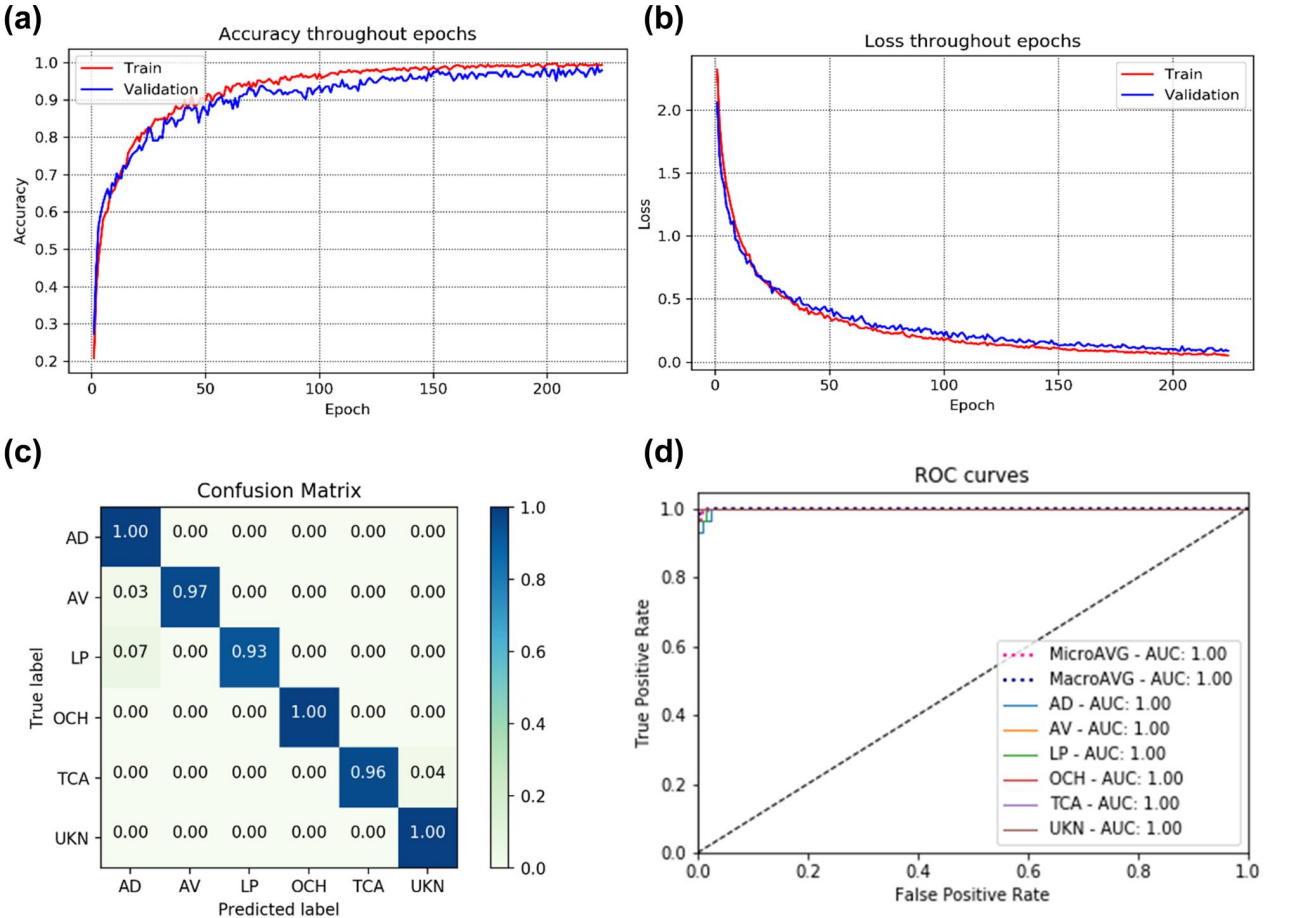
Summary of multiclass classifier using both image and patient information: (a) training and validation accuracy, (b) training and validation loss, (c) confusion matrix and (d) receiver operator characteristic (ROC) curve

Training the model using clinical images only resulted 94.2% training and 88.3% validation accuracy at the 45th epoch, with the lowest validation loss of 0.306, as demonstrated in Figure 7. This model correctly classified 138 images out of 157 test images resulting test accuracy of 87.9% and kappa score of 0.86 (Table 4). On the other hand, the model which was trained using both clinical images and patient information achieved 99.5% training and 97.9% validation accuracy at the 214th epoch, with the lowest validation loss of 0.084, as demonstrated in Figure 8. This model correctly classified 153 images of the 157 test datasets resulting a test accuracy of 97.5% and kappa score of 0.976 (Table 5).

**TABLE 4:** Test performance of the multiclass classifier model trained using images only

Skin condition	Precision (%)	Recall (%)	F1‐score (%)	Kappa score	Accuracy (%)
Acne vulgaris	87	90	89	**0.86**	**87.9**
Atopic dermatitis	85	77	81		
Lichen planus	88	79	84		
Onychomycosis	100	100	100		
Tinea capitis	100	96	98		
Unknown	69	90	78		
Average	**88.2**	**88.7**	**89.8**		

*Note*: The values in bold show the average test performances of the model.

**TABLE 5:** Test performance of the multiclass classifier model trained on both clinical image and patient information

Skin condition	Precision (%)	Recall (%)	F1‐score (%)	Kappa score	Accuracy (%)
Acne vulgaris	91	100	95	**0.976**	**97.5**
Atopic dermatitis	100	97	98		
Lichen planus	100	93	96		
Onychomycosis	100	100	100		
Tinea capitis	100	96	98		
Unknown	95	100	98		
Average	**97.7**	**97.7**	**97.5**		

*Note*: The values in bold show the average test performance of the model.

**FIGURE 9:**
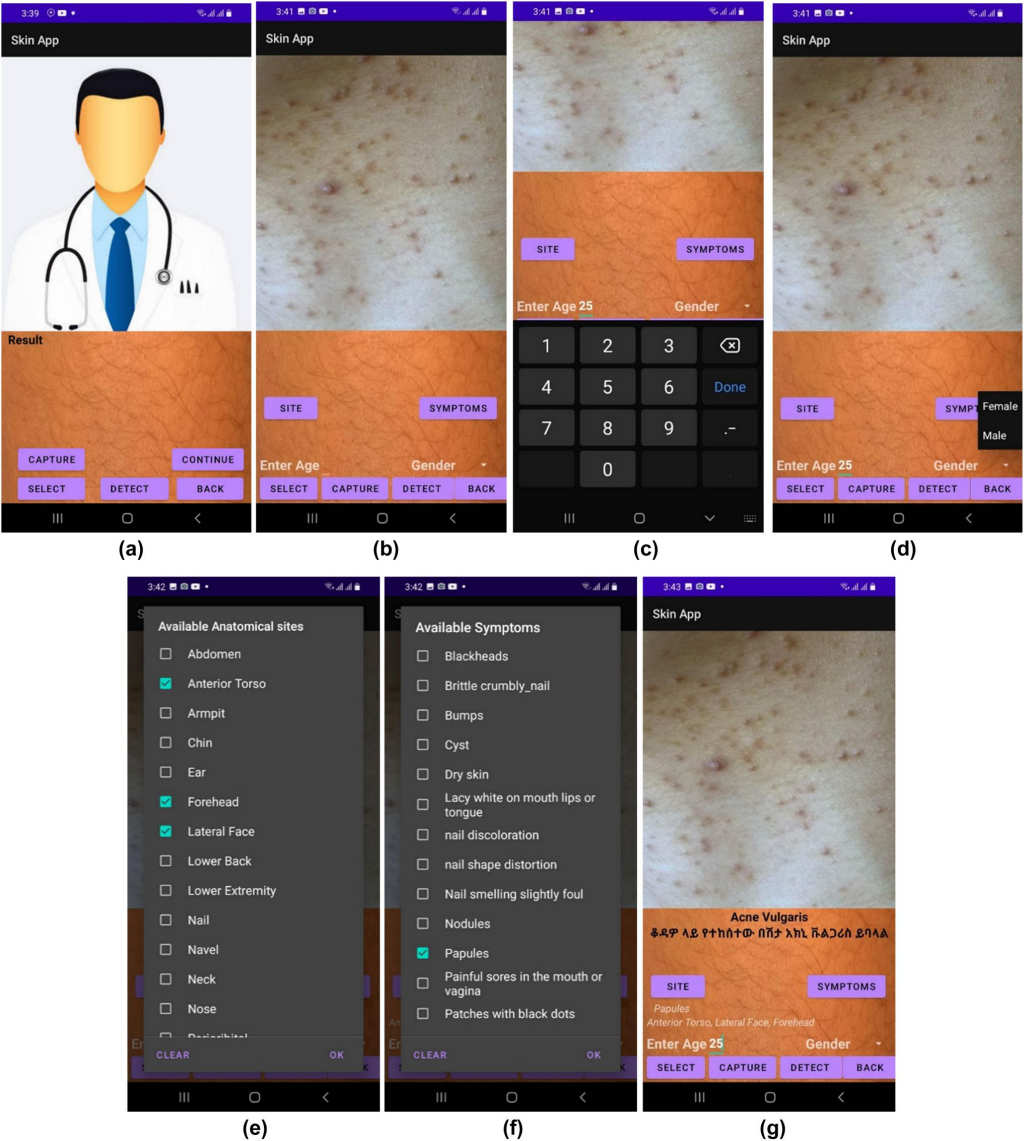
An android application used for diagnosing skin diseases using clinical image and patient information. (a) Home page of the App. (b) Image Capturing. (c) Age information. (d) Gender selection. (e) Anatomical site selection. (f) Symptom list selection. (e) Predicted skin disease

An android application has been also developed for ease of using the proposed automatic skin diseases diagnosis system using smartphones. Through the developed application, the user can capture skin images, enter age and select anatomical sites, gender and symptoms to identify the type of skin disease. After loading the user can diagnose the skin condition by hitting detect button. The first window enables to diagnose the skin condition as healthy or abnormal. Next, if the result is abnormal hitting the continue button pops up another window to diagnose the five‐skin condition. If the disease was out of the five classes the model returns Unknown. Figure 9 demonstrates the developed android application.

## 4 DISCUSSION

Skin diseases are the fourth leading cause of non‐fatal disease burden^3^ affecting almost 900 million people worldwide^2^ which can cause serious psychological problem including depression, frustration and even suicidal ideation.^13^ The shortage of sophisticated diagnostic devices and dermatologists and even general practitioners in developing countries make things worse and hurdle the service delivery. Moreover, the common diagnostic techniques including visual inspection, laboratory test, imaging and biopsy tests are tedious and require experience and excellent visual perception. Computer‐aided diagnosis systems have a potential to revolutionize the current disease diagnosis techniques enabling optimal treatment planning.

The aim of this study was to design and develop a smartphone based automatic skin disease diagnosis method using skin images and patient information including age, gender, anatomical site of the disease and symptom list. A total of 1880 skin images of top five diseases were collected from the Southwest of Ethiopia (Dr. Gerbi Medium Clinic, Jimma), Eastern Amhara, and Afar region (Boru‐Meda General Hospital), using different smartphone cameras with the corresponding patient information. The type of skin diseases was different from place to place, but the selected five diseases were common, in average, in these places.

From the collected data, tinea capitis was common in children with the age range of 0–9 years, rare on 10–19 years and absent above the age of 20 years. On the other hand, atopic dermatitis was found in all age ranges, but it was found to be common within the age range of 0–39 years. Moreover, acne vulgaris was common in the age range of 10‐49 years, especially teens and adults were found to be more vulnerable in the age range of 10–29 years. Even though, onychomycosis was found to occur in evenly people with the age of 0–59 years. In addition, lichen planus was also common between the ages 20–49 and rarely found in teens adults and elderly people. This is demonstrated in Figure 3. Figure 4 demonstrates gender wise distribution of the skin disease from the collected data. Acne vulgaris and onychomycosis were more common on females than males, and tinea capitis was more common on males than females.

A Mobilenet‐v2 model was trained using the collected data, first to identify normal skin from abnormal using images only and then uses a combinations of skin images and patient information to identify the type of skin disease.

MobileNetv2 models are a simple model with a smaller number of parameters which is suitable for mobile and embedded vision applications. It is works better than other state of the art pre‐trained models with latency, size and accuracy. It is also suitable for smartphone devices which have limited storage capacity and processing speed. Different state of the art pre‐trained models were including SqueezeNet, ShuffleNet, efficientnetB0, NASNetMobile with varying number of parameters were considered. However, MobileNet‐v2 model was selected for our purposes due to its lower number of parameters and best accuracy.

All images and patient information were pre‐processed prior to model training. The colour cast resulted from illumination variation was removed and the actual colour of the images were restored by applying shades of grey colour constancy algorithm.^35^ After data pre‐processing, the clinical images along with the corresponding patient data were split to 80%, 10% and 10% for training, validation and testing, respectively. Data augmentation was applied by using image transformation technique to increase the number of training data. The weighted loss function based on labels frequency was applied to tackle class imbalance issue. A pre‐trained Mobilent‐v2 model was selected and fine‐tuned for binary and multiclass classification for training, since it is the best model for mobile devices and resource limited environment.

For binary classification, best result was achieved by applying a learning rate of 0.0001, sigmoid activation function as classifier, and cross entropy loss as a loss function. The average accuracy, precision, recall, F1‐score and kappa score achieved were 98.3%, 98.5%, 98.5%, 98.5% and 0.97, respectively. The multiclass classifier classifies the input clinical image in to six different skin conditions. The unknown class was added as a sixth class to reduce false‐positive result. As a result, image lesions out of the five classes were classified as unknown. The best result was achieved by applying learning rate of 0.00001, SoftMax activation function as a classifier and weighted cross‐entropy loss as a loss function. The model was tested using unseen datasets and evaluated using a variety of performance metrices. The average accuracy, precision, recall, F1‐score and kappa score were 87.9%, 88.2%, 88.7%, 89.8% and 0.86, respectively, using images only (Table 4). An improved performance was achieved using a combination of images and patient information and an average accuracy, precision, recall, F1‐score, and kappa score of 97.5%, 97.7%, 97.7%, 97.5% and 0.976, respectively were acquired after testing the model on unseen dataset (Table 5). Using the combined image and patient information increase the classification accuracy of the model by 9.6%.

Since there are about 3000 and more skin diseases,^14^ different researchers proposed machine learning and deep learning based diagnosis systems for specific types of diseases.^22^, ^23^, ^24^, ^25^, ^26^, ^27^, ^28^, ^29^ Our study focused on top five diseases that are common in Ethiopia. Even though, the dataset used and the types of diseases considered were slightly different, the current work, achieved significantly improved overall accuracy compared to the studies^22^, ^23^, ^24^, ^25^, ^26^, ^27^, ^28^, ^29^ by incorporating patient and clinical information. A user‐friendly android application will also enable non‐expert users to identify the skin diseases using their smartphones. The developed system has a potential to be used as a decision support system for physicians, general practitioners, and patients. We acknowledge the symptoms of disease include in this study can be erroneous as patient's perception of clinical observations may deviate from clinical judgement especially during self‐diagnosis.

## 5 CONCLUSION

In this study, a smartphone based automatic diagnosis of five common skin diseases, is proposed based on a deep learning technique using clinical image and patient clinical information, and an average accuracy, precision, recall, F1‐score, and kappa score of 97.5%, 97.7%, 97.7%, 97.5% and 0.976, respectively, were achieved. The results demonstrate that, the developed system provides excellent diagnosis performance for the five skin diseases. The developed diagnostic system has a potential to be used as a decision support system for dermatologists, general practitioners, health practitioners in rural areas and patients in the diagnosis of skin disease.

## CONFLICT OF INTEREST

The authors declare no conflicts of interest.

## AUTHOR CONTRIBUTIONS

**K. A. Muhaba:** Conceptualization; Data curation; Formal analysis; Funding acquisition; Investigation; Methodology; Software; Validation; Visualization; Writing – original draft; Writing – review & editing. **K. Dese:** Investigation; Methodology; Supervision; Writing – review & editing. **T. M. Aga:** Data curation; Formal analysis; Investigation; Validation; Writing – review & editing. **F. T. Zewdu:** Data curation; Investigation; Resources; Validation; Writing – review & editing. **G. L. Simegn:** Conceptualization; Formal analysis; Methodology; Project administration; Supervision; Validation; Writing – original draft; Writing – review & editing.

## Data Availability

The datasets used and/or analyzed during the current study are available from the corresponding author on reasonable request.
